# Altered Ex Vivo NLRP3 Inflammasome Activation Is Associated with 28-Day Mortality in Septic Patients

**DOI:** 10.3390/v15122419

**Published:** 2023-12-13

**Authors:** Rémy Coudereau, Guillaume Monneret, Anne-Claire Lukaszewicz, Bénédicte F. Py, Laurent Argaud, Martin Cour, Frank Bidar, Morgane Gossez, Fabienne Venet

**Affiliations:** 1 Hospices Civils de Lyon, Edouard Herriot Hospital, Immunology Laboratory, 69437 Lyon, France; remy.coudereau@chu-lyon.fr (R.C.); guilaume.monneret@chu-lyon.fr (G.M.); morgane.gossez@chu-lyon.fr (M.G.); 2 EA 7426 “Pathophysiology of Injury-Induced Immunosuppression” (Université Claude Bernard Lyon 1-Hospices Civils de Lyon-bioMérieux), Joint Research Unit HCL-Biomérieux, 69437 Lyon, France; anne-claire.lukaszewicz@chu-lyon.fr (A.-C.L.); frank.bidar@chu-lyon.fr (F.B.); 3 Hospices Civils de Lyon, Edouard Herriot Hospital, Anesthesia and Critical Care Medicine Department, 69437 Lyon, France; 4 CIRI, Centre International de Recherche en Infectiologie, Univ Lyon, Inserm U1111, Université Claude Bernard-Lyon 1, CNRS, UMR5308, ENS de Lyon, 69007 Lyon, France; benedicte.py@inserm.fr; 5 Hospices Civils de Lyon, Edouard Herriot Hospital, Medical Intensive Care Department, 69002 Lyon, France; laurent.argaud@chu-lyon.fr (L.A.); martin.cour@chu-lyon.fr (M.C.)

**Keywords:** sepsis, COVID-19, inflammation, NLRP3 inflammasome, ASC speck, caspase-1, flow cytometry

## Abstract

Sepsis is a life-threatening organ dysfunction caused by a dysregulated response to infection. In this context, the aberrant activation of the NLRP3 inflammasome has been documented mostly through the measurement of increased plasmatic concentrations of IL-1β and IL-18. At the cellular level, contradictory results have been published. However, no study has comprehensively monitored NLRP3 inflammasome activation at the basal level and after ex vivo reactivation of whole blood monocytes and neutrophils focusing on ICU patients with bacterial and viral sepsis, including a longitudinal analysis. Thus, we conducted a prospective longitudinal study, examining NLRP3 inflammasome functionality in COVID-19 ICU patients (*n* = 15) and bacterial septic shock patients (*n* = 17) during the first week of ICU hospitalization, compared with healthy donors. Using two whole-blood flow cytometry assays, we detected ASC speck-positive monocytes (i.e., monocytes presenting the polymerization of ASC proteins) and activated caspase-1 in polymorphonuclear cells as read-outs, both at baseline and following nigericin stimulation, a drug that forms pores and activates the NLRP3 inflammasome. Our findings showed that, at baseline and regardless of the type of infection, patients exhibited reduced ASC speck-positive monocytes and decreased activated caspase-1 in PMN compared to healthy volunteers. This decrease was prominent at day 0. Following nigericin stimulation, this reduction was also observed and persisted throughout the first week of hospitalization, irrespective of the cellular population or parameter being considered. Notably, at day 0, this diminished activation and response to stimulation of NLRP3 was associated with a higher 28-day mortality rate. Consequently, our observations highlighted a concurrent decline in both basal expression and ex vivo activation of the NLRP3 inflammasome in circulating myeloid cells from patients with bacterial and viral sepsis in association with increased mortality.

## 1. Introduction

Sepsis is a life-threatening organ dysfunction caused by a dysregulated host response to various infections (e.g., bacterial, viral) [1]. Sepsis represents the leading cause of deaths in intensive care units (ICU) and has been recognized as a global health priority by the WHO [2]. The immune response to sepsis is a complex and dynamic phenomenon associating pro- and anti-inflammatory immune responses [3]. Dysregulation of either the pro- or the anti-inflammatory responses could lead to mortality in cases of sepsis.

Inflammasomes are central to the innate immune response during sepsis. Inflammasomes are intracellular multiprotein complexes that activate upon recognition of danger signals. Their activation involves a two-step process: “priming” and “activation”, which results in the conversion of pro-caspase-1 to its active form, the caspase-1 [4]. Of these, the NLRP3 inflammasome is the most extensively studied. It is formed of a sensor (NLRP3) that recruits an adaptor protein called ASC (apoptosis-associated speck-like protein with a caspase activating and recruitment domain). Upon activation, ASC polymerizes, forming the ASC specks, which leads to the recruitment and activation of caspase-1. This, in turn, cleaves pro-IL-1β and pro-IL-18 into their active pro-inflammatory forms IL-1β and IL-18 [4].

Aberrant activation of the NLRP3 inflammasome has been linked to the physiopathology of various inflammatory diseases. These include auto-inflammatory periodic fever syndromes such as NLRP3-associated auto-inflammatory diseases (NLRP3-AID) [5]. Furthermore, its role has also been identified in conditions like cancer, diabetes, gout, cardiovascular [6], and neurological disorders [7]. In cases of sepsis, including bacterial sepsis and viral conditions like COVID-19 (coronavirus disease-2019), aberrant activation of the NLRP3 inflammasome has also been documented mostly through the measurement of increased plasmatic concentrations of IL-1β and IL-18 [8,9,10,11].

At the cellular level, contradictory results have been published with reports describing either enhanced NLRP3 inflammasome activation in monocytes and polymorphonuclear cells (PMN) from COVID-19 or septic patients [10,12], while other articles rather described decreased responses [13,14]. Several technical and clinical aspects could explain these discrepancies. Indeed, read-outs and techniques for NLRP3 inflammasome monitoring differed between studies (whole blood versus purified cells, ASC specks versus caspase-1 activation or IL-1β release). Secondly, NLRP3 inflammasome activation was monitored either at the basal level or after cell stimulation ex vivo. Finally, studies frequently mixed patients with increasing severity (mild, severe, or critically ill).

In total, so far no study has comprehensively monitored NLRP3 inflammasome activation at the basal level and after ex vivo reactivation of whole blood monocyte (ASC-speck formation) and neutrophils (activated caspase-1 staining) focusing on ICU patients with bacterial and viral sepsis, including a longitudinal analysis. This represented the aim of the current study.

## 2. Materials and Methods

### 2.1. Study Population

Patients admitted to two ICUs of Edouard Herriot Hospital (Hospices Civils de Lyon, Lyon, France) were included in two prospective observational studies: RICO (REA-IMMUNO-COVID) [15] and IMMUNOSEPSIS4 [16]. Regarding the RICO cohort, COVID-19 ICU patients over 18 years of age with a confirmed diagnosis of SARS-CoV2 infection were enrolled. This project was approved by the Institutional Review Board for ethics (“Comité de Protection des Personnes Ile de France 1”, n° RCB: 2020-A01079-30). This study is registered at the French Ministry of Research and Teaching (#DC-2008-509), at the Commission Nationale de l’Informatique et des Libertés, and on clinicaltrials.gov (NCT04392401). Regarding the IMMUNOSEPSIS4 cohort, patients over 18 years of age were enrolled for septic shock based on Sepsis-3 criteria [1]. This project was approved by the Institutional Review Board for ethics (“Comité de Protection des Personnes Ouest II”, n° RCB: 2019-A00210-57). This study is registered at the French Ministry of Research and Teaching (#DC-2008-509), at the Commission Nationale de l’Informatique et des Libertés, and on clinicaltrials.gov (NCT02803346). For both cohorts, patients’ samples and clinical data were collected three times after ICU admission: within 48 h after admission (day 0: D0), between D3 and D5 (D3), and between D7 and D9 (D7). Both ethylene diamine tetraacetic acid (EDTA) and heparinized blood samples were utilized in this study. EDTA-anticoagulated samples were used for studying immunological parameters. Briefly, CD4+ T cells immunophenotyping was performed on an automated volumetric flow cytometer from Beckman Coulter (Aquios CL) as previously described [17]. Monocyte HLA-DR (mHLA-DR) expression measurement was performed using the Anti-HLA-DR/Anti-Monocyte Quantibrite assay (BD Biosciences, San Jose, USA) as previously described [18]. Heparin-anticoagulated samples were used for ASC speck detection in monocytes and caspase-1 staining in PMN. Oral information and non-opposition to inclusion in the study were mandatory and recorded in patients’ clinical files for both studies.

Peripheral blood from healthy donors (HD) was provided by the “Etablissement Français du Sang (EFS)” from Lyon. Clinical data were recorded and a written non-opposition to the use of donated blood for research purposes was obtained from the HD.

### 2.2. Reagents

The following antibodies were used: CD45-PB, CD14-APC (both from Beckman Coulter, Brea, CA, USA, clones J33 and RMO52, respectively), and anti-ASC-PE (from Biolegend, San Diego, CA, USA, clones HASC-71). The following reagents were used: phosphate-buffered saline (PBS) (Eurobio Scientific, Luxembourg, Luxembourg), RPMI HEPES (Eurobio, Les Ulis, France), a FAM FLICA™ Caspase-1 Kit (Biorad, Hercules, CA, USA), versalyse (Beckman Coulter), nigericin (InvivoGen, San Diego, CA, USA), water for injection (Aguettant, Lyon, France), and a Cytofix/Cytoperm™ Fixation/Permeabilization Kit, stain buffer (both from BD Bioscience, Franklin Lakes, NJ, USA).

### 2.3. NLRP3 Activation Monitoring in Whole Blood Myeloid Cells

Two whole-blood assays are currently available to explore the ex vivo reactivation functionality of the NLRP3 inflammasome. The first method focuses on the formation of ASC specks in monocytes, an assay developed by our lab. Briefly, this method can detect the polymerization of ASC proteins into specks, using flow cytometry. The second technique monitors caspase-1 activation in PMN using a fluorescent probe specific to its active state, and the resultant signal is then detected in flow cytometry.

#### 2.3.1. Monocytes ASC Staining in Whole Blood

**Figure 1:**
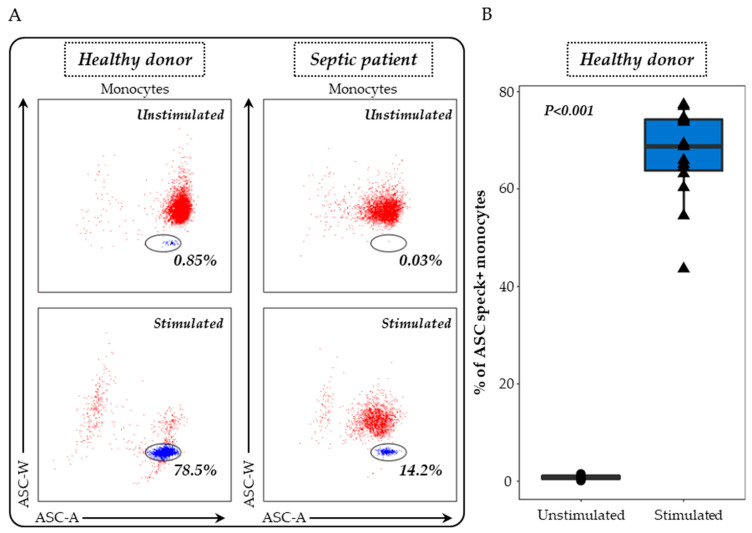
Evaluation of ASC speck-positive monocytes in whole blood. (**A**) Speck formation was assessed using a bi-parametric ASC-area (ASC-A)/ASC-width (ASC-W) histogram gated on monocytes (CD14+ cells highlighted in red). ASC speck-positive cells display diminished ASC-width (highlighted in blue within the circle) in comparison with non-specking cells. The results from a representative healthy donor and a septic patient, both unstimulated (upper panel) and post-stimulation with 10 μg/mL nigericin for 30 min (lower panel), are presented. (**B**) The ASC speck formation in whole blood monocytes was examined in healthy donors (*n* = 15) under two conditions: unstimulated (black) and after stimulation with 10 μg/mL nigericin for 30 min (blue). The nonparametric Wilcoxon paired test was used to assess variations among healthy donors under both unstimulated and stimulated conditions.

ASC specks were detected on heparinised whole blood as previously described [19]. Briefly, 100 μL of whole heparin blood was first diluted in 100 µL of lysis solution (Versalyse) for 10 min to remove red blood cells. Cells were then washed with PBS for 10 min and then centrifuged. After supernatant removal, cells were re-suspended in 500 μL of PBS and incubated with or without nigericin at 10 μg/mL for 30 min, at 37 °C. Cells were washed with stain buffer, centrifuged and supernatant removed. Cells were then labelled with 5 μL of PB-labelled anti-CD45 antibody and 5 μL of APC-labelled anti-CD14 antibody, incubated for 15 min and washed with stain buffer for 6 min at 300 g. A permeabilization step was performed using Cytoperm. After two washing steps with diluted Perm/Wash buffer, cells were labelled with 5 μL of PE-labelled anti-ASC antibody for 20 min. A final wash was performed with Perm/Wash buffer, supernatant removed and cells re-suspended in 300 μL of PBS. Samples were run on a FACS ARIA II cytometer (BD Bioscience, Franklin Lakes, NJ, USA) and results expressed as percentages of ASC speck-positive monocytes among total monocytes. The gating strategy and representative examples are shown in Figure 1.

#### 2.3.2. Caspase-1 Staining in PMN

**Figure 2:**
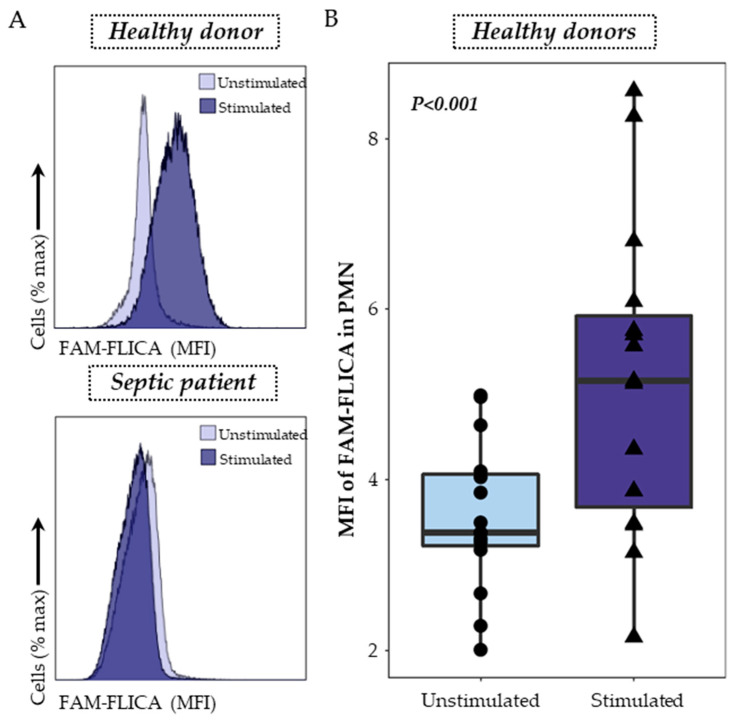
Detection of activated caspase-1 in whole blood neutrophils. (**A**) Activated caspase-1 was monitored using a monoparametric FAM-FLICA histogramgated on PMN. The overlays, representing the median fluorescence intensity (MFI) of FAM-FLICA in unstimulated conditions (light purple) and after stimulation with 10 μg/mL nigericin for 30 min (dark purple), are shown as examples for both a healthy volunteer (upper panel) and a septic patient (lower panel). (**B**) The activation of caspase-1 in whole blood neutrophils was examined in healthy donors (*n* = 15) under two conditions: unstimulated (light purple) and after stimulation with 10 μg/mL nigericin for 30 min (dark purple). The nonparametric Wilcoxon paired test was used to assess variations among healthy donors under both unstimulated and stimulated conditions.

Caspase-1 staining was performed using a FAM FLICA™ Caspase-1 Kit following the manufacturer’s instructions. Briefly, 100 μL of whole heparin blood was first diluted in 100 µL of RPMI HEPES and then incubated with or without nigericin at 10 μg/mL for 30 min at 37 °C in the dark. The FLICA solution was diluted to form the 30X solution, and 10 µL was added and incubated for 30 min at 37 °C in the dark. Cells were then washed with 1 mL of RPMI HEPES for 10 min at 37 °C and centrifuged. After supernatant removal, cells were re-suspended in 100 µL of staining buffer and then labelled with 2 μL of PB-labelled anti-CD45 antibody, and incubated 10 min at room temperature, in the dark. Cells were then incubated with 1 mL of lysing solution (Versalyse) for 10 min at room temperature in the dark and washed with PBS before being analyzed using flow cytometry. Samples were run on a Navios flow cytometer (Beckman Coulter, Brea, CA, USA) and results expressed as Median Fluorescent Intensity (MFI) of FAM-FLICA among PMN. The gating strategy and representative examples are shown in Figure 2.

### 2.4. Statistical Analysis

Continuous data are presented as medians and interquartile ranges [Q1–Q3], categorical data are presented as numbers and percentages. Statistical analyses were performed using the non-parametric Mann–Whitney U test comparing data in COVID-19 or in septic patients versus healthy donors; the nonparametric Wilcoxon paired test was used to assess variations in healthy donors between different stimulation conditions and correlations were calculated using the Spearman correlation test. Data were analyzed with R (version 4.2.1, Boston, MA, USA).

## 3. Results

### 3.1. Patients Characteristics

We included 32 septic patients: 15 with COVID-19 hospitalized in the ICU and 17 with bacterial septic shock, along with 15 healthy donors. Table 1 details their clinical and biological characteristics. Most patients fit the typical profile of septic individuals, being older males with high severity scores. Generally, these patients displayed reduced mHLA-DR expression and a tendency toward CD4+ T cell lymphopenia, indicative of the immunosuppressed state commonly observed in sepsis. Information pertaining to different etiologies and details about healthy donors are presented in the Supplementary Table S1.

### 3.2. Detection of ASC Specks in Monocytes and Activated Caspase-1 in PMN as Indicators of NLRP3 Inflammasome Activation

The formation of ASC specks in monocytes served as our primary measure to evaluate both the basal or in vivo activation of the NLRP3 inflammasome and its ex vivo response to nigericin stimulation. For this purpose, after doublets exclusion, monocytes were identified among whole blood cells based on CD45 and CD14 co-expressions. Monocytes exhibiting ASC speck formation were identified through the specific signal observed on the bi-parametric ASC-area (ASC-A)/ASC-width (ASC-W) histogram (blue cells, Figure 1A) [19]. While the percentage of ASC+ monocytes was very low at the basal state in unstimulated cells from HD, a large increase in the proportion of ASC+ monocytes was detected after nigericin stimulation (Figure 1B). Representative examples in one healthy donor and one septic patient are shown.

Activated caspase-1 detection in PMN was the second assay explored, using a specific fluorescent probe, the FAM-FLICA. PMN were gated on a bi-parametric CD45/side scatter histogram and the MFI of the FAM-FLICA was analyzed in the previously gated cells. Representative examples in one healthy donor and one septic patient are shown (Figure 2A). In healthy volunteers’ blood, nigericin stimulation was associated with a significant increase in the median fluorescence intensity of FMA-FLICA in PMN (Figure 2B).

### 3.3. Basal Expression and Ex Vivo Reactivation of the NLRP3 Inflammasome in Septic Patients

**Figure 3:**
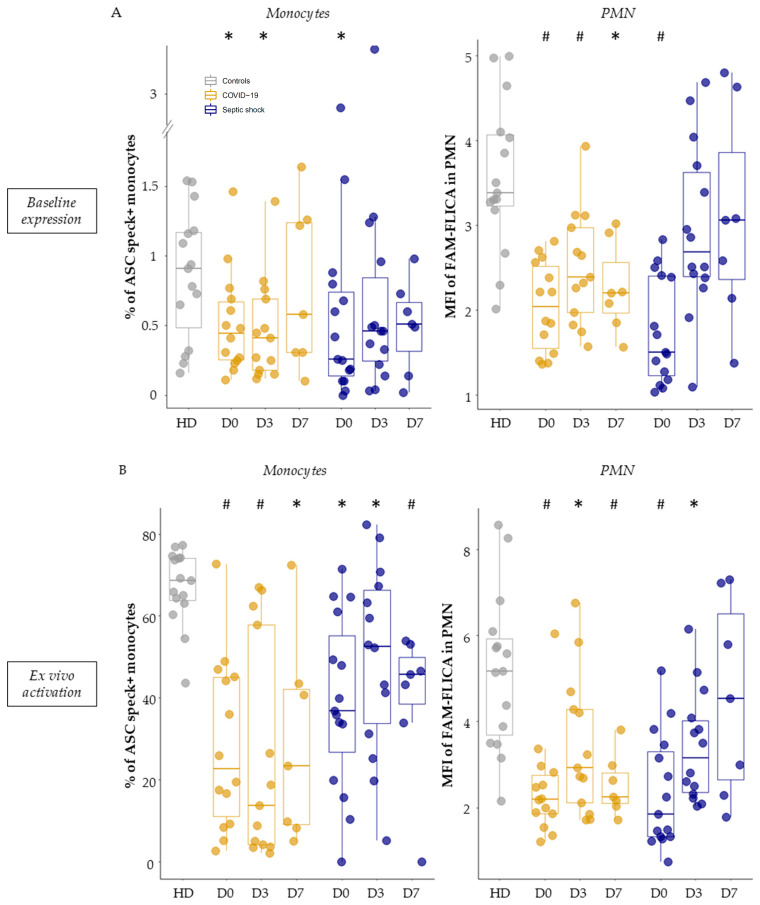
Measurement of basal NLRP3 inflammasome activity and its ex vivo reactivation capacity post-stimulation in myeloid populations. Percentages of ASC speck-positive monocytes (left panel) and median fluorescence intensities (MFI) of FAM-FLICA in polymorphonuclear cells (PMN) (right panel) were measured in healthy volunteers (HD, in grey, *n* = 15), as well as in patients with COVID-19 (in gold, *n* = 15) or septic shock (in blue, *n* = 17) during their first week of hospitalization. This was conducted under basal conditions (**A**) and following ex vivo stimulation with nigericin at 10 µg/mL for 30 min (**B**). These assessments were conducted within the first 48 h of hospitalization for COVID-19 patients (D0, *n* = 14), between day 3–5 (D3, *n* = 13), and between day 6–9 (D7, *n* = 7), as well as for patients with septic shock (D0, *n* = 15; D3, *n* = 14; D7, *n* = 7). Results are presented as individual values and boxplots. The non-parametric Mann–Whitney test was used to compare values between controls and patients at any specific time-point. * *p* < 0.05, # *p* < 0.001.

Under basal conditions (without stimulation, thus reflecting in vivo activity), we observed a decreased inflammasome activation in monocytes and PMN from septic patients. Indeed, patients exhibited a reduction in the percentage of ASC speck-positive monocytes compared to healthy volunteers (Figure 3A, left panel). This reduction was especially pronounced on day 0 (median = 0.45% for COVID-19 patients and 0.26% for patients with bacterial septic shock). Similarly, when analyzing the MFI of FAM-FLICA in PMN under basal conditions, there was a marked decrease in activated caspase-1 in patients compared to healthy volunteers (Figure 3A, right panel). This decrease was consistently observed throughout the monitoring period for COVID-19 ICU patients and was evident on day 0 for patients with bacterial septic shock.

**Figure 4:**
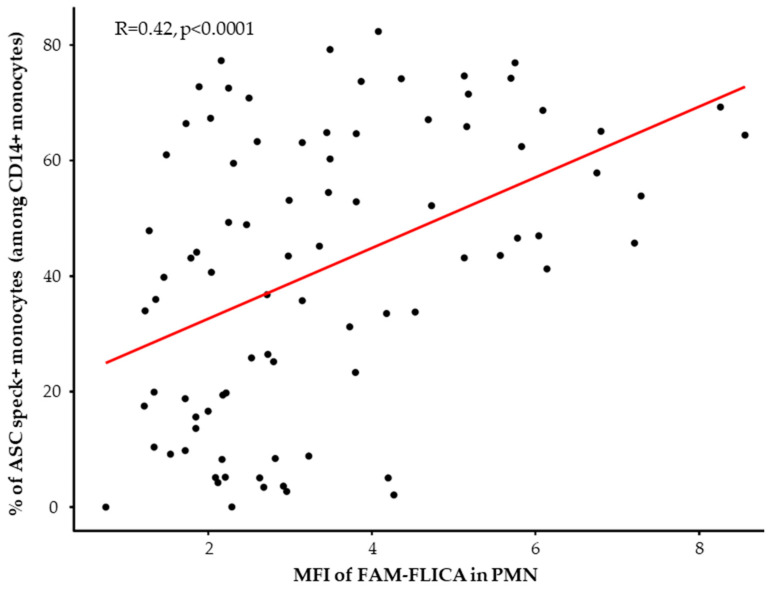
Correlation between the percentages of ASC speck-positive monocytes and median fluorescence intensities (MFI) of FAM-FLICA in neutrophils in healthy donors (HD) (*n* = 15) and septic patients at all assessed time points (*n* = 70) after ex vivo stimulation using 10 µg/mL nigericin for 30 min. Correlation was calculated using Spearman correlation test.

When looking at the reactivation abilities of the NLRP3 inflammasome after ex vivo stimulation with nigericin, we similarly observed a decreased NLRP3 inflammasome activation in monocytes and PMN from patients. Indeed, we observed a consistent reduction in the ability of monocytes from all septic patients to form ASC specks during the entire first week in the ICU compared to healthy volunteers (Figure 3B, left panel). Parallel insights came from studying caspase-1 activation post-stimulation in PMN (Figure 3B, right panel). Notably, this decrease appeared to be less marked by day 7 in patients with bacterial septic shock. Finally, we analyzed the correlation between the percentages of ASC speck+ monocytes and MFI of activated caspase-1 in PMN after ex vivo stimulation in HD and patients (Figure 4). We observed a positive significant correlation between ASC speck formation in monocytes and activated caspase-1 in PMNs.

In total, whether under basal conditions or after ex vivo stimulation, the NLRP3 inflammasome showed diminished activation and reactivation abilities in both monocytes and PMN of patients with viral and bacterial sepsis, which appeared to be more marked upon admission.

### 3.4. Association between NLRP3 Inflammasome Activation and 28-Day Mortality in Septic Patients

Given the similar NLRP3 inflammasome activation and reactivation profiles observed in both ICU COVID-19 patients and those with bacterial septic shock, we combined the two cohorts for survival analyses.

**Figure 5:**
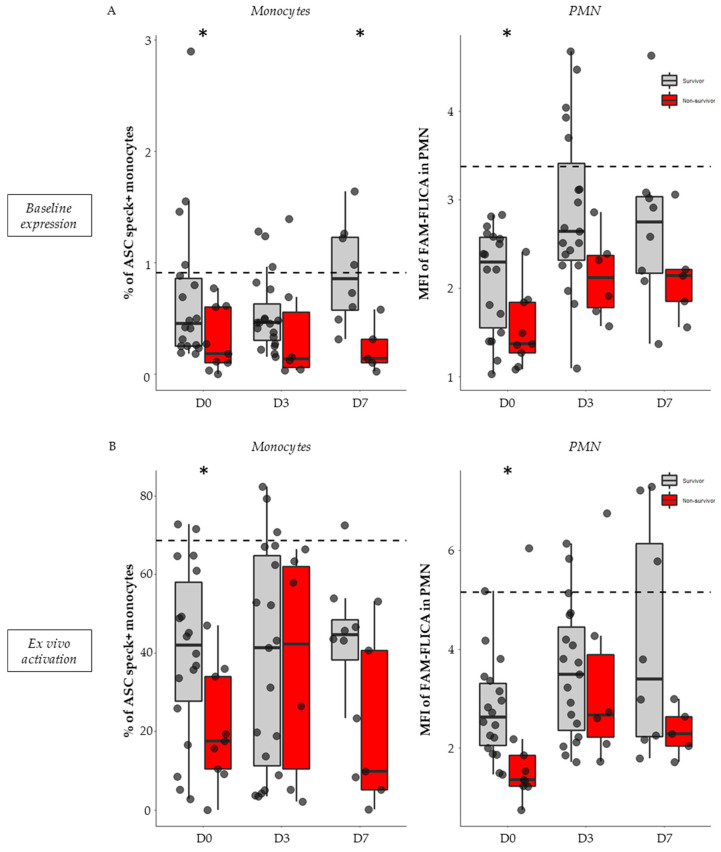
Association of NLRP3 inflammasome basal expression and ex vivo reactivation with 28-day mortality in septic patients. Percentages of ASC speck-positive monocytes and MFI of FAM-FLICA in PMN were assessed in septic patients (*n* = 32) within the first 48 h of hospitalization (D0, *n* = 27), between days 3–5 (D3, *n* = 26), and between days 6–9 (D7, *n* = 13). This was conducted under basal conditions (**A**) and following ex vivo stimulation with nigericin at 10 µg/mL for 30 min (**B**). Patients were stratified based on their 28-day status: survivors (*n* = 21, in grey) or non-survivors (*n* = 9, in red). Missing data points relate to patients with unknown 28-day statuses (*n* = 2). Results are expressed as individual values and boxplots. The dotted black line indicates the median value observed in healthy volunteers for the respective parameter. The Mann–Whitney non-parametric test was used for comparison between survivors and non-survivors. * *p* < 0.05.

In non-stimulated conditions (i.e., basal activation level), 28-day non-survivors exhibited a diminished proportion of ASC speck-positive monocytes and decreased activated caspase-1 in PMN compared with survivors. This trend was observed during the first week after ICU admission but was most notable on day 0 both for ASC+ monocytes (0.18% vs. 0.45%, *p* = 0.040, Figure 5A, left panel) and FAM-FLICA in PMNs (1.37 vs. 2.29, *p* = 0.025, Figure 5A, right panel).

Similarly, after ex vivo stimulation, a decrease in NLRP3 reactivation capacity was observed in monocytes and PMN from non-survivors compared with survivors. This was most significant on day 0 both for ASC speck formation in monocytes (18.45% vs. 42%, *p* = 0.041, Figure 3B, left panel) and caspase-1 activation in PMNs (1.36 vs. 2.62, *p* = 0.0043, Figure 5B, right panel).

In total, whether under basal conditions or after ex vivo stimulation, the NLRP3 inflammasome diminished activation and reactivation abilities of both monocytes and PMN was more pronounced in non-survivors compared with survivor patients with viral and bacterial sepsis.

## 4. Discussion

The immune response in patients with sepsis is a complex interplay between both pro- and anti-inflammatory processes. Many studies have highlighted robust activation of the NLRP3 inflammasome, mainly through the assessment of its systemic activation via IL-1β and IL-18 levels, which are linked to mortality [9,10,11,20]. However, at the cellular and functional level, the findings are contradictory, with some studies showing activation in monocytes and PMN [10,12], while others rather suggested a decreased activation [13,14]. Therefore, our goal was to simultaneously explore both the basal activity of the NLRP3 inflammasome and its ex vivo reactivation using whole blood functional tests suitable to clinical exploration studies. We assessed speck formation in monocytes and caspase-1 in neutrophils, with these two tests being considered as reference tests when looking at NLRP3 activation. We conducted this investigation in critically ill patients with bacterial or viral sepsis (COVID-19) during their first week of hospitalization.

Overall, the present study offers evidence of a widespread deficiency in NLRP3 inflammasome activation and reactivation abilities, affecting both monocytes and PMN. The study highlights several important findings: (i) a baseline reduction of NLRP3 inflammasome markers in both monocytes and PMN, particularly evident upon patients’ admission; (ii) a significant and persistent decrease in the capacity of monocytes and neutrophils to reactivate upon ex vivo stimulation; and (iii) the most severe impairments in functions, such as speck formation and caspase-1 activation, were observed in patients who ultimately succumbed within 28 days.

On one hand, the present results appear to contradict previous studies that have demonstrated an increase in NLRP3 parameters in both COVID-19 and septic patients. Some studies have reported an elevation in the proportion of monocyte forming specks, increased caspase-1 activity, and heightened production of IL-1β and IL-18 from peripheral blood mononuclear cells (PBMCs) in COVID-19 patients [11,12]. One study revealed a significant increase in the percentage of monocytes showing positivity for ASC specks from Day 6 onwards [8], although no substantial difference was observed in PMN. On the other hand, there is a substantial body of literature describing a deficiency in the NLRP3 inflammasome that aligns with our results. For instance, a decrease in caspase-1 activity at baseline in the PMN of hospitalized COVID-19 patients has been observed [14]. In septic patients, another study revealed the suppression of IL-1β release at the onset of sepsis in both survivors and non-survivors [21]. Notably, this suppression persisted and remained low in non-survivors over time. In a different study, the authors found that the production of IL-1β by peripheral blood mononuclear cells was compromised after stimulation by various stimuli, and the extent of this impairment was directly linked to the severity of the patients’ conditions [13]. Additionally, the authors showed a reduction in caspase-1 activation through western blot analysis. This aligns with the “NLRP3 immunocompromised” proposed by Martinez-Garcia and colleagues who reported a decline in both IL-1β production and the formation of ASC specks in septic patients [22]. This phenotype was characterized by an extremely high mortality rate of 80% rate among septic patients.

Various factors may account for the observed differences. Firstly, it is important to distinguish between the systemic activation of the NLRP3 inflammasome, as indicated by plasma concentrations of IL-1β and IL-18, and cellular evaluation, which examines parameters such as the formation of ASC specks or caspase-1 activity. Furthermore, variations in results could be attributed to the use of different types of samples. For instance, the utilization of purified PBMCs after ficoll purification may introduce priming which may induce bias in NLRP3 cellular activation monitoring in patients (in contrast to the use of whole blood). Secondly, patient severity plays a crucial role. These studies were conducted on diverse groups of septic patients, each with varying levels of severity, including infection, sepsis, and septic shock. Notably, Giamarellos-Bourboulis et al. demonstrated that the decrease in IL-1β production, following ex vivo stimulation, was linked to the disease severity [13]. Thirdly, understanding the dynamics of a condition seems to be more crucial than relying on a single measurement at a specific moment.

Overall, the current study provides consistent observations, as we report on two distinct cellular types and two different methods for monitoring NLRP3 activation in native whole blood, all yielding similar conclusions. Furthermore, the results were obtained from a relatively homogeneous group of critically ill septic patients, characterized by similar severity in the ICU. Importantly, a longitudinal follow-up was conducted over the first week of their ICU hospitalization, involving multiple measurements for each patient. This approach allowed us to illustrate that patients who do not survive are consistently those who exhibit low activation levels. Despite studies reporting on elevated plasmatic levels of IL-1β and IL-18, it seems that the cells can no longer activate their NLRP3 inflammasome. This raises questions about the origin of the cells producing these cytokines. One hypothesis suggests that it could be due to compartmentalization, involving the recruitment of inflammatory cells to the infection site while, in parallel, systemic feedback mechanisms inhibit immune cells outside the infection site [23,24]. Alternatively, pyroptosis could be the main actor, releasing cytokines but leaving cells unable to respond to additional challenges [25,26]. These results bring to mind the phenomenon of endotoxin tolerance [13,27], which is well-established in sepsis.

It is important to acknowledge the limitations of this study. The relatively small size of our cohort underscores the need for additional clinical studies with a larger number of participants to validate our initial findings. Furthermore, future research should delve into the potential impact of gender on immune responses, a topic recognized in the existing literature but not addressed in this study due to the predominance of male participants in our cohort. Finally, it should be noted that the time of disease onset, although challenging to determine, differs between bacterial and viral etiologies concerning admission to the ICU. Therefore, adequately representing this aspect would necessitate further investigation.

## 5. Conclusions

Collectively, present results and past observations agree on the occurrence of a deficiency in NLRP3 inflammasome activation and reactivation abilities in sepsis. This alteration seems to be associated with unfavorable outcomes. Therefore, comprehensive studies in mice are essential to better understand the pathophysiological mechanisms responsible for this deactivation of the NLRP3 inflammasome, as it may open new therapeutic strategies to tackle the deleterious immunosuppression observed in septic patients.

## Figures and Tables

**Table 1:** Demographic, clinical, and immunological data for septic patients. Continuous data are presented as medians and interquartile ranges (Q1–Q3). For clinical parameters, categorical data are presented as numbers of cases and percentages among the total population in brackets. SAPS II (simplified acute physiology score II) and SOFA (sequential organ failure assessment) scores were calculated at admission. Absolute CD4+ T cell count (expressed as cells/µL) as well as mHLA-DR (expressed as numbers of anti-HLA-DR antibodies bound per monocyte, AB/C) were calculated on day 3. Reference values from our routine lab: mHLA-DR: 13,500–45,000 AB/C, CD4+: 336–1126 cells/μL.

Parameters	Septic Patients (*n* = 32)
Age at admission (years)	64 (61–69)
Gender–Male *n* (%)	25 (78)
Main admission category n (%) Medical Surgical	22 (69) 10 (31)
SAPSII score at admission	38 (33–46)
SOFA score at admission	6 (3–10)
Charlson score	1 (0–3)
Infection diagnosis n (%) Microbiology Surgery Other	26 (81) 3 (9) 3 (9)
Microbiologically documented n (%) Bacilli gram − Cocci gram + SARS-CoV-2	7 (22) 4 (12) 15 (46)
Respiratory dysfunction Mechanical ventilation n (%) PaO_2_/FiO_2_	18 (56) 177 (86–279)
Site of primary infection n (%) Pulmonary Abdominal Other	16 (50) 10 (31) 4 (12)
Follow-up n (%) 28-day non survivors Secondary nosocomial infections	9 (28) 3 (9)
Immunological parameters (at Day 3) mHLA-DR (AB/C) CD4+ T cell count (cells/µL)	5914 (4748–7954) 339 (140–553)

## Data Availability

Data are available from corresponding author upon reasonable request.

## Supplementary Materials

The following are available online at https://www.mdpi.com/article/10.3390/v15122419/s1, Table S1: Demographic, clinical, and immunologic data for viral septic (COVID-19) and bacterial septic shock patients.

## References

[1] Singer M., Deutschman C.S., Seymour C.W., Shankar-Hari M., Annane D., Bauer M., Bellomo R., Bernard G.R., Chiche J.-D., Coopersmith C.M. (2016). The Third International Consensus Definitions for Sepsis and Septic Shock (Sepsis-3). JAMA.

[2] Reinhart K., Daniels R., Kissoon N., Machado F.R., Schachter R.D., Finfer S. (2017). Recognizing Sepsis as a Global Health Priority—A WHO Resolution. N. Engl. J. Med..

[3] Hotchkiss R.S., Monneret G., Payen D. (2013). Sepsis-induced immunosuppression: From cellular dysfunctions to immunotherapy. Nat. Rev. Immunol..

[4] Groslambert M., Py B.F. (2018). Spotlight on the NLRP3 inflammasome pathway. J. Inflamm. Res..

[5] Booshehri L.M., Hoffman H.M. (2019). CAPS and NLRP. J. Clin. Immunol..

[6] Sharma B.R., Kanneganti T.-D. (2021). NLRP3 inflammasome in cancer and metabolic diseases. Nat. Immunol..

[7] Holbrook J.A., Jarosz-Griffiths H.H., Caseley E., Lara-Reyna S., Poulter J.A., Williams-Gray C.H., Peckham D., McDermott M.F. (2021). Neurodegenerative Disease and the NLRP3 Inflammasome. Front. Pharmacol..

[8] Cui J., Oehrl S., Ahmad F., Brenner T., Uhle F., Nusshag C., Rupp C., Funck F., Meisel S., Weigand M.A. (2020). Detection of In Vivo Inflammasome Activation for Predicting Sepsis Mortality. Front. Immunol..

[9] Nasser S.M.T., Rana A.A., Doffinger R., Kafizas A., Khan T.A., Nasser S. (2023). Elevated free interleukin-18 associated with severity and mortality in prospective cohort study of 206 hospitalised COVID-19 patients. Intensive Care Med. Exp..

[10] Rasmussen G., Idosa B.A., Bäckman A., Monecke S., Strålin K., Särndahl E., Söderquist B. (2019). Caspase-1 inflammasome activity in patients with Staphylococcus aureus bacteremia. Microbiol. Immunol..

[11] Rodrigues T.S., de Sá K.S.G., Ishimoto A.Y., Becerra A., Oliveira S., Almeida L., Gonçalves A.V., Perucello D.B., Andrade W.A., Castro R. (2021). Inflammasomes are activated in response to SARS-CoV-2 infection and are associated with COVID-19 severity in patients. J. Exp. Med..

[12] Bertoni A., Penco F., Mollica H., Bocca P., Prigione I., Corcione A., Cangelosi D., Schena F., Del Zotto G., Amaro A. (2022). Spontaneous NLRP3 inflammasome-driven IL-1-β secretion is induced in severe COVID-19 patients and responds to anakinra treatment. J. Allergy Clin. Immunol..

[13] Giamarellos-Bourboulis E.J., van de Veerdonk F.L., Mouktaroudi M., Raftogiannis M., Antonopoulou A., Joosten L.A.B., Pickkers P., Savva A., Georgitsi M., van der Meer J.W. (2011). Inhibition of caspase-1 activation in Gram-negative sepsis and experimental endotoxemia. Crit. Care.

[14] Courjon J., Dufies O., Robert A., Bailly L., Torre C., Chirio D., Contenti J., Vitale S., Loubatier C., Doye A. (2021). Heterogeneous NLRP3 inflammasome signature in circulating myeloid cells as a biomarker of COVID-19 severity. Blood Adv..

[15] Venet F., Cour M., Rimmelé T., Viel S., Yonis H., Coudereau R., Amaz C., Abraham P., Monard C., Casalegno J.S. (2021). Longitudinal assessment of IFN-I activity and immune profile in critically ill COVID-19 patients with acute respiratory distress syndrome. Crit. Care.

[16] Venet F., Textoris J., Blein S., Rol M.-L., Bodinier M., Canard B., Cortez P., Meunier B., Tan L.K., Tipple C. (2022). Immune Profiling Demonstrates a Common Immune Signature of Delayed Acquired Immunodeficiency in Patients With Various Etiologies of Severe Injury. Crit. Care Med..

[17] Gossez M., Malcus C., Demaret J., Frater J., Poitevin-Later F., Monneret G. (2017). Evaluation of a novel automated volumetric flow cytometer for absolute CD4+ T lymphocyte quantitation. Cytom. Part B Clin. Cytom..

[18] Demaret J., Walencik A., Jacob M.-C., Timsit J.-F., Venet F., Lepape A., Monneret G. (2013). Inter-laboratory assessment of flow cytometric monocyte HLA-DR expression in clinical samples. Cytom. B Clin. Cytom..

[19] Coudereau R., Gossez M., Py B.F., Henry T., Lukaszewicz A.-C., Monneret G., Venet F. (2022). Monitoring NLRP3 Inflammasome Activation and Exhaustion in Clinical Samples: A Refined Flow Cytometry Protocol for ASC Speck Formation Measurement Directly in Whole Blood after Ex Vivo Stimulation. Cells.

[20] Rogers A.J., Guan J., Trtchounian A., Hunninghake G.M., Kaimal R., Desai M., Kozikowski L.-A., DeSouza L., Mogan S., Liu K.D. (2019). Association of Elevated Plasma Interleukin-18 Level With Increased Mortality in a Clinical Trial of Statin Treatment for Acute Respiratory Distress Syndrome. Crit. Care Med..

[21] Weighardt H., Heidecke C.D., Emmanuilidis K., Maier S., Bartels H., Siewert J.R., Holzmann B. (2000). Sepsis after major visceral surgery is associated with sustained and interferon-gamma-resistant defects of monocyte cytokine production. Surgery.

[22] Martínez-García J.J., Martínez-Banaclocha H., Angosto-Bazarra D., de Torre-Minguela C., Baroja-Mazo A., Alarcón-Vila C., Martínez-Alarcón L., Amores-Iniesta J., Martín-Sánchez F., Ercole G.A. (2019). P2X7 receptor induces mitochondrial failure in monocytes and compromises NLRP3 inflammasome activation during sepsis. Nat. Commun..

[23] Cavaillon J.M., Adib-Conquy M., Cloëz-Tayarani I., Fitting C. (2001). Immunodepression in sepsis and SIRS assessed by ex vivo cytokine production is not a generalized phenomenon: A review. J. Endotoxin Res..

[24] Cavaillon J.-M., Annane D. (2006). Compartmentalization of the inflammatory response in sepsis and SIRS. J. Endotoxin Res..

[25] Dai Z., Liu W.-C., Chen X.-Y., Wang X., Li J.-L., Zhang X. (2023). Gasdermin D-mediated pyroptosis: Mechanisms, diseases, and inhibitors. Front. Immunol..

[26] Wang Y., Liu Y., Liu Q., Zheng Q., Dong X., Liu X., Gao W., Bai X., Li Z. (2020). Caspase-1-Dependent Pyroptosis of Peripheral Blood Mononuclear Cells Is Associated with the Severity and Mortality of Septic Patients. BioMed Res. Int..

[27] Allantaz-Frager F., Turrel-Davin F., Venet F., Monnin C., De Saint Jean A., Barbalat V., Cerrato E., Pachot A., Lepape A., Monneret G. (2013). Identification of biomarkers of response to IFNg during endotoxin tolerance: Application to septic shock. PLoS ONE.

