# Supplementary material for: Altered Ex Vivo NLRP3 Inflammasome Activation Is Associated with 28-Day Mortality in Septic Patients
Source: Viruses. 2023 Dec 13;15(12):2419. doi: 10.3390/v15122419 (PMC10748301; doi:10.3390/v15122419)
Supplement: Supplementary file 1 [file viruses-15-02419-s001.zip › viruses-2731929-supplementary.pdf]

Altered ex vivo NLRP3 Inflammasome Activation is Associated with 28-Day Mortality in septic patients

Rémy Coudereau[1,2], Guillaume Monneret[1,2], Anne-Claire Lukaszewicz[2,3], Bénédicte F Py[4], Laurent Argaud[5], Martin Cour[5], Frank Bidar[2,3], Morgane Gossez[1,4], Fabienne Venet[1,4*]

Supplementary Material

Parameters	**COVID-19** (n=15)	**Septic shock** (n=17)	**Healthy donors** (n=15)
Age at admission (years)	64 [51-71]	65 [61-69]	44 [33-55]
Gender - Male *n (%)*	11 (73)	14 (82)	5 (33)
**Main admission** *n (%)*			
Medical	15 (100)	7 (41)	
Surgical	0 (0)	10 (59)	
SAPSII score	34 [26-39]	45.5 [38-56]	
SOFA score	3 [1-5]	10 [7-12]	
Charlson score	0 [0-1.5]	2 [1-6]	
**Infection diagnosis** n (%)			
Microbiology	15 (100)	12 (70)	
Surgery	0 (0)	3 (18)	
Other	0 (0)	2 (12)	
**Microbiologicaly documented** *n (%)*			
Bacilli gram -	0 (0)	7 (64)	
Cocci gram +	0 (0)	4 (36)	
Sars-CoV-2	15 (100%)	0 (0)	
**Respiratory dysfunction**			
Mechanical ventilation *n (%)*	3 (20)	15 (88)	
PaO_2/FiO_2	76.5 [67-93]	248 [177-294]	
**Site of primary infection** *n (%)*			
Pulmonary	15 (100)	1 (6)	
Abdominal	0 (0)	11 (69)	
Other	0 (0)	4 (25)	
**Follow-up** *n (%)*			
28-day non survivors	5 (33)	4 (27)	
Secondary nosocomial infections	1 (7)	2 (12)	
**Immunologic parameters** (at Day 3)			
mHLA-DR (AB/C)	7300 [5299-8842]	5161 [3613-6475]	19570 [18146-29700]
Absolute CD4+ T cell count (cells/µL)	166 [126-462]	343 [174-681]	675 [542-734]

Supplementary Table. 1 Demographic, clinical, and immunologic data for viral septic (COVID-19) and bacterial septic shock patients. Continuous data are presented as medians and interquartile ranges [Q1–Q3]. For clinical parameters, categorical data are presented as numbers of cases and percentages among the total population in brackets. SAPS II (simplified acute physiology score II) and SOFA (sequential organ failure assessment) scores were calculated at admission. Absolute CD4+ T cell count (expressed as cells/µL) was calculated on day 3 as well as mHLA-DR (expressed as numbers of anti-HLA-DR antibodies bound per monocyte, AB/C). Reference values from our routine lab: mHLA-DR: 13500-45000 AB/C, CD4+: 336–1126 cells/µl.
